# Could H5N1 bird flu virus be the cause of the next human pandemic?

**DOI:** 10.3389/fmicb.2024.1477738

**Published:** 2024-10-08

**Authors:** Giorgio Palù, Pier Francesco Roggero, Arianna Calistri

**Affiliations:** Department of Molecular Medicine, University of Padua, Padua, Italy

**Keywords:** avian influenza viruses, H5N1, pandemic, viral evolution, chaos theory

## 1 Introduction

Highly pathogenic type A avian influenza (HPAI) viruses have been affecting numerous wild and domestic avian species worldwide over the past decades and continue to circulate, causing significant damage to the poultry industry (CDC, 2024a). Since 2021, numerous regions worldwide, including Europe, have faced a severe panzootic outbreak of the HPAI H5N1 virus, clade 2.3.4.4b (Koopmans et al., 2024). This outbreak has caused the death or culling of hundreds of millions of farmed and wild birds, making it one of the largest avian influenza outbreaks on record (CDC, 2024a). It has also led to substantial economic losses and severely disrupted poultry supply chains (Banyard et al., 2024). The H5N1 subtype was first detected at a commercial goose farm in China in 1996 (Watanabe et al., 2013; Tiwari et al., 2024) and later spread to a wide range of bird species (CDC, 2024a). Over time, the virus evolved, and by 2024, H5N1 clade 2.3.4.4b had developed into 16 genotypes, with the dominant G1 genotype being detected across multiple countries. This clade has exhibited an unprecedented range of host infections and pathogenicity, with infections reported in over 90 species of wild and domestic birds, as well as more than 199 mammalian species, including cats, foxes, minks, harbor seals and sea lions (Caserta et al., 2024). The virus has even reached polar regions, causing the death of a polar bear in the Arctic and affecting elephant and fur seals as well as penguins in Antarctica (Banyard et al., 2024). Particularly intriguing and worrying are the recent cases in dairy cattle, as bovine species were known to be poorly susceptible to influenza A viruses (IAV) and were previously reported not susceptible to H5N1 (Sreenivasan et al., 2019). In 2024, however, H5N1 clade 2.3.4.4b was found to infect cattle of a number of American farms, with confirmed transmission to humans in four cases (CDC, 2024a,b; Oguzie et al., 2024; Burrough et al., 2024). Since 1996, there have been occasional spillovers of H5N1 also to humans. These spillovers have only occurred when there was close contact at the avian-mammal-human interface. Additionally, there have been only a few reported cases of probable but not sustained human-to-human transmission (CDC, 2024a; Ungchusak et al., 2005; Wang et al., 2008). Notably, on the 6^th^ of September 2024, CDC reported the case of a patient from Missouri, USA, positive for an H5 type IAV. This patient had no known immediate exposure to animals, and no transmission was detected among close contacts (CDC, 2024c). In 2024, a total of 15 human cases of H5 were documented in the USA. With the exception of the Missouri case, all others were linked to contact with infected animals—four involving infected dairy cows and the rest involving infected poultry. As for cows, subjects infected by H5N1 from dairy cattle (hereafter referred to as Cow-H5N1) exhibited mild symptoms, primarily conjunctivitis, malaise, and low-grade fever (Webby and Uyeki, 2024; CDC, 2024a). Human infections with HPAI H5 viruses typically have high fatality rates, averaging around 50%. It remains unclear whether Cow-H5N1 viruses are inherently less pathogenic in humans compared to other HPAI strains, or if factors such as the route of infection and/or viral load contribute to the milder symptoms observed.

What is certain is that the recent detection of HPAI H5N1 in dairy cattle represents a significant cross-species transmission, highlighting the virus adaptability and its ability to infect mammals, particularly domestic ruminants, across multiple farms. Although current influenza surveillance shows no unusual human influenza activity, the zoonotic potential of the currently circulating H5N1 remains a concern. The possibility of a new pandemic caused by this virus is, then, a subject of ongoing debate (Burki, 2024; Dye and Barclay, 2024; Koopmans et al., 2024; The Lancet, 2024; Zhai et al., 2024). The prospect of this virus acquiring mutations that enable efficient human-to-human transmission is especially alarming. However, we believe that H5N1 is unlikely to become pandemic at least in the near future. This perspective is grounded on three key points, which we will detail in the following sections:

(a) The virus has not yet demonstrated efficient airborne transmission. (b) The virus still needs to acquire certain genetic traits known to facilitate widespread transmission to human populations. (c) The chaos logistic map indicates a low probability for this virus to adapt to the human populations.

Nonetheless, given the widespread nature of the current outbreaks, the risk of a future pandemic, while uncertain, cannot be entirely ruled out.

## 2 Cow-H5N1 has not yet demonstrated efficient airborne transmission

The detection of mammal-to-mammal transmission of avian-origin influenza viruses (AIV) remains rare and depends on both genetic and phenotypic adaptations to the new hosts. The H5N1 virus and its lineages, including the clade that is currently causing the influenza outbreak in American cattle, have been circulating in nature for nearly 30 years, infecting millions of animals. Since 1996, this virus has undergone continuous evolution, gaining the ability to efficiently spread among wild and domestic avian species and spill over into several mammalian species. What is particularly fascinating about the recent outbreak in cattle is that bovines have largely remained unaffected by IAV in the past (Sreenivasan et al., 2019). Indeed, IAV has rarely been detected in bovine species, suggesting that these mammals were not vulnerable hosts for the virus. This view persisted until the emergence of influenza D viruses, for which cattle are considered the primary reservoir (Ruiz et al., 2022). Certain bovine factors were proposed to possess anti-influenza properties, potentially explaining the resistance of cattle to IAV infection (Sreenivasan et al., 2019). Among these factors (extensively reviewed in Sreenivasan et al., 2019), specific serum components and secretory proteins were shown to influence IAV infection, primarily by interacting with components of the immune system. Additionally, bovine lactoferrin was reported to act as a broad-spectrum anti-influenza compound (Ammendolia et al., 2012; Shin et al., 2005). Finally, interferon-α-induced bovine Mx protein shows a potent antiviral activity due to peculiar N-terminal motifs (Garigliany et al., 2012). On one hand, these findings make it very unlikely for cows to become efficient reassortment vessels that facilitate the emergence of IAV with pandemic potential. On the other hand, it is crucial to understand what has enabled this specific clade of H5N1 to infect and spread among cattle.

Current data suggest that the H5N1 outbreak in dairy cattle stemmed from a single introduction of an HPAI H5 virus into this new host (Worobey et al., 2024a,b). The virus may have been transmitted to cattle farms by infected birds or mammals. However, the precise events that led to the introduction of HPAI H5 viruses into cattle remain unclear (Neumann and Kawaoka, 2024). In a recent study, Eisfeld et al. (2024) investigated the pathogenicity and transmission routes of an H5N1 clade 2.3.4.4b virus, isolated from milk during the 2024 outbreak in New Mexico. Using mice as an experimental model, those Authors demonstrated that ingestion of infected milk led to rapid viral dissemination in both respiratory and non-respiratory organs. It was reported that cows' mammary glands express avian-type (α-2,3) sialic acid receptors (Nelli et al., 2024) and more recently even the human-type (α-2,6) receptor (Kristensen et al., 2024), which could enable H5N1 shedding in bovine milk. In agreement, it has been shown that bovine breast functions as a site for sustained viral replication, a condition that might favor the generation of a virus with human tropism and pandemic potential (Mallapaty, 2024). These findings confirm earlier experiments (Mitchell et al., 1953; Paquette et al., 2015) and support the hypothesis that cow-to-cow transmission mainly occurs via milking equipment. Vertical transmission from infected lactating mice to their offspring was also observed, raising concerns about the possibility of similar transmission from cows to calves (Eisfeld et al., 2024).

Experiments based on ferrets were used to assess whether Cow-H5N1 could be transmitted via respiratory droplets among mammals. Although one contact ferret seroconverted, no viral RNA or particles were detected, indicating inefficient transmission (Eisfeld et al., 2024). This result is consistent with findings from the CDC (2024d), which demonstrated a limited respiratory droplet transmission between ferrets infected with a Cow-H5N1 isolated from a human case linked to contact with infected dairy cattle occurred in Texas, USA. Although the CDC's study did demonstrate transmission in 1 out of 3 ferret pairs, overall, these two investigations show that Cow-H5N1 remains inefficient at mammal-to-mammal transmission via respiratory routes. Indeed, for efficient airborne transmission to occur, the virus would need to acquire several phenotypic adaptations, including a shift in hemagglutinin (HA) binding specificity from avian to human receptors and enhanced replication in mammalian cells.

## 3 Cow-H5N1 still needs to acquire certain genetic traits known to facilitate widespread transmission to the human population

The growth of AIV is largely species-restricted due to the viral envelope protein hemagglutinin (HA), which facilitates viral entry into target cells by recognizing the cell receptor (Long et al., 2019). The limited number of H5N1 human cases reported so far, even among close contacts, can be partly attributed to the low affinity of H5 for the α2-6 linked sialic acid human receptors, which contributes to a significant species barrier. Interestingly, Eisfeld and colleagues observed that a Cow-H5N1 isolated from milk in New Mexico, USA, displayed binding affinity for both α-2,3 avian and α-2,6 human receptors (Eisfeld et al., 2024). This finding suggests that one key trait necessary for human-to-human transmission is already present in the virus. Accordingly, a recent study focusing on the genetic characterization and phylogenetic analysis of clade 2.3.4.4b H5N1 viruses obtained from dairy cattle, cats and wild birds in Texas (Hu et al., 2024) demonstrated that all the viruses investigated from bovines and felines shared specific amino acid residues in the HA sequence (e.g., 137A, 158N, 160A). These residues have been shown to enhance the affinity of IAV for human-type receptors (Yamada et al., 2006; Gao et al., 2009). However, critical mutations associated with mammalian host adaptation and increased transmission (Gao et al., 2009; Suttie et al., 2019; Bordes et al., 2023; Hatta et al., 2007; Kong et al., 2019; Kamal et al., 2015) were notably absent in the same isolates. Furthermore, in the viruses obtained from cattle no amino acid substitutions were identified associated to an improved replication or transmission in mammals. This result indicates that the overall risk to human health remains relatively low at present. Furthermore, in two preprint studies Cow-H5N1 isolates were reported to preferentially bind to avian receptors (Chopra et al., 2024; Santos et al., 2024). Additionally, the viral HA retained all the amino acids that confer avian receptor specificity, with no evidence supporting an acquired ability to bind to the human receptor (Chopra et al., 2024). Thus, continued monitoring for any changes in receptor binding specificity appears to be essential. Indeed, a successful species jump would require several significant mutations in the HA gene. Specifically, more than one amino acid change would be needed in the highly conserved receptor binding domain (RBD) of H5, particularly between amino acids 128 and 138 (Imai et al., 2012; Xiong et al., 2013). In terms of virus adaptation and long-term evolution, it is noteworthy that out of the eighteen HAs identified in different animal species, the only pandemic influenza viruses affecting humans have been those with H1, H2, or H3 types (Nabel and Fauci, 2010; Kumar et al., 2018).

A remarkable feature of the 2.3.4.4b lineage is the pairing of H5 with a full-length (long-stalk) neuraminidase (NA), unlike earlier H5N1 viruses, which typically exhibited stalk deletions (short-stalk). This pairing may have contributed to the virus ability to infect a broad range of species and could play a role in its transmission between mammals (Moratorio et al., 2024). The role of the long stalk in facilitating virus adaptation to mammals is undoubtedly a key area for further research, also in the context of vaccination strategies.

On the other hand, it is important to recognize that IAV can evolve rapidly within their hosts following infection. Indeed, in one of the human cases involving direct contact with infected dairy cattle, the amino acid substitution E627K in the PB2 subunit of the viral polymerase complex was identified, a mutation that is absent in isolates from cows (CDC, 2024d). Of note, the virus carrying the E627K mutation was the one used by CDC in the study discussed above (CDC, 2024e) focused on the airborne transmission of H5N1 in ferrets. This mutation might be the reason of the higher transmission efficiency found in the CDC report with respect to the work carried on by Eisfeld et al. (2024). This finding confirms that the E627K mutation in the PB2 sequence may favor H5N1 spreading in mammals, humans included (Gabriel et al., 2013; Long et al., 2019), and highlights the potential for viral adaptation especially if spillover events and circulation in mammals are not efficiently controlled.

## 4 H5N1 is unlikely to become pandemic according to the chaos theory logistic map

The chaos theory logistic map (May, 1976; Fernández-Díaz, 2024) is a mathematical model known for exhibiting chaotic behavior under specific conditions. The model is formally expressed by the following equation (May, 1976):


$$\begin{array}{l} x\;\left(n+1\right)=r\;*x\;\left(n\right)\;*\;\left[1-x\left(n\right)\right] \end{array}$$

where the growth rate “*r*” reflects the combined effects of reproduction and density-dependent mortality (e.g., starvation) on the size of a population. The population size *x* (*n*) stays within the range [0, 1] if *r* is between 0 and 4. If *r* is below 1, the population will go extinct. Higher *r* values can lead to either a stable population size or fluctuations across a range of values. For *r* > 3.57, the population grows chaotically.

We recently applied the logistic map to understand the dynamics of pandemics and epidemics caused by emerging viruses (Roggero et al., 2023; Calistri et al., 2024). In particular, we focused on the growth of infection cases for three coronaviruses recently emerged in the human population—SARS-CoV-1, MERS-CoV, and SARS-CoV-2—as well as for the Ebola virus responsible for the 2014 West Africa epidemic. We demonstrated that the *r* value reflects the intrinsic biological characteristics of an emerging virus. Indeed, this value mirrors the virus initial growth and spread within the population and its potential to establish a lasting relationship with the new host (Roggero et al., 2023). More in particular, an emerging virus can only spread and adapt if its initial *r* is associated with chaotic growth, as seen with SARS-CoV-2. Since this adaptation involves specific viral genome mutations, we speculated that an initial chaotic growth facilitates the acquisition of these beneficial genomic changes, whereas other types of growth do not (Roggero et al., 2023).

With these premises and using available data from November 1997 to March 2024, we determined that the *r* value for H5N1 circulation in humans was 0.56 over the first 5 years of virus circulation, which then decreased to 0.18, resulting in a weighted mean value of 0.26 over the 27 years of observation. Even when restricting the analysis to the period from January 2020 to September 2024 to better capture the current situation, which differs significantly from previous conditions, we found an *r* value of 0.01102. Thus, the *r* value for H5N1 circulating in humans has consistently remained below 1. Since an *r* value of < 1 is associated with population extinction (Fernández-Díaz, 2024; Roggero et al., 2023), we conclude that H5N1 is unlikely to cause a human pandemic.

## 5 Conclusion

We acknowledge that some information about H5N1 remains incomplete, which could affect the virus potential to become pandemic, making it difficult to precisely predict the likelihood of such an event. Specifically, there is a lack of data on the serostatus of individuals in close contact with known human or animal cases of avian flu and on the percentage of people who may have been asymptomatically or mildly infected by H5N1. In addition, it has been previously shown that 2009 H1N1 influenza virus infection elicits cross reactive immunity to avian H5N1 viruses (Mahallawi et al., 2013), clade 2.3.4.4b H5N1 included (Daulagala et al., 2024). It would be interesting to analyze whether this applies also to the currently circulating H5N1 and thus whether previous exposure to 2009 H1N1 could have an impact on the spreading of H5N1 in the human population.

What we currently know is that Cow-H5N1 has still limited airborne transmission. At the moment, the main risk for humans, in addition to occupational exposure, comes from the consumption of raw milk, while commercial pasteurization eliminates infectious virus from milk (Spackman et al., 2024). In addition, the absence of replicating H5N1 in beef meet has been demonstrated (USDA, 2024).

Although Cow-H5N1 possesses certain features that could promote infection and transmission among mammals, it still lacks some critical genetic traits known to be required for efficient spread in humans. Thus, in our view, supported by the chaos theory logistic map, it is unlikely that H5N1 will become a pandemic agent, at least in the near future.

Finally, circulating H5N1 viruses respond to currently available influenza virus drugs. Furthermore, there are already approved pre-pandemic H5N1 vaccines as well as candidate vaccine viruses (CVVs) on the pipelines (Focosi and Maggi, 2024). In July 2024 WHO announced a project to develop an mRNA H5N1 vaccine for humans (Anderer, 2024). While these vaccines and CVVs may only partially match a potential future pandemic strain, they can still serve as an interim solution to protect high-risk individuals until new vaccines become accessible.

Although we expect these factors to mitigate the impact of H5N1 on public health, the sustained circulation of H5N1 in mammals, even in species like dairy cattle, which were previously considered poorly susceptible to IAV, increases the likelihood of the virus evolving to adapt to humans, with unpredictable characteristics in terms of pathogenicity and transmission potential.

Hence, we strongly advocate for more extensive tracing and testing of influenza virus presence at the human-animal interface using a One Health approach. This should be accompanied by a continuous, global exchange of information among veterinary, environmental, and human virologists.
